# Egg desensitization is achieved effectively and safely through a low maintenance dose protocol

**DOI:** 10.1016/j.jacig.2025.100547

**Published:** 2025-07-31

**Authors:** Diana Toscano-Rivero, Nofar Kimchi, Wei Zhao, Jana Abi-Rafeh, Danbing Ke, Duncan Lejtenyi, Liane Beaudette, Christine McCusker, Bruce D. Mazer, Moshe Ben-Shoshan

**Affiliations:** a Division of Experimental Medicine, Department of Medicine, McGill University and the Research Institute of the McGill University Health Centre (RI-MUHC), Montreal, Quebec, Canada; b Division of Allergy and Immunology, Department of Pediatrics, Montreal Children’s Hospital, Montreal, Quebec, Canada

**Keywords:** Food allergy, egg allergy, skin prick test, DBPCFC, oral food challenge, oral immunotherapy, low dose, allergic reactions

## Abstract

**Background:**

Egg allergy is a common IgE-mediated food allergy in children. Oral immunotherapy (OIT) reduces allergic reactions via gradual allergen exposure. Although high maintenance doses (1-6 g egg protein) are often used, they carry higher risks of adverse reactions. Evidence on the safety and effectiveness of lower-dose egg OIT (E-OIT) remains limited.

**Objective:**

We sought to determine whether a low dose of 300 mg E-OIT is safe and effective for desensitization.

**Methods:**

Twenty-two participants were recruited from the Montreal Children’s Hospital; 20 were randomized to an immediate-treatment group or an observation group (egg avoidance for 1 year before OIT). Cumulative tolerated dose (CTD), Gal d 1– and Gal d 2–specific IgE (sIgE) and –specific IgG4 (sIgG4), and skin prick test responses were measured at baseline, postescalation, and maintenance. Adverse events were recorded throughout the study.

**Results:**

At baseline, CTD did not differ between groups (treatment: median, 14.1 mg; observation: 20.1 mg; *P* = 1). After 1 year of egg avoidance, CTD in the observation group remained low (32.3 mg; *P* = .06). Following E-OIT, the treatment group showed a significant increase in CTD to 2000 mg at postescalation (*P* = .004) and 6000 mg at the exit double-blind, placebo-controlled food challenge (*P* = .009). The observation group, after crossover, reached 1000 mg postescalation (*P* = .02) and 5000 mg at exit (*P* = .04). Adverse reactions occurred in 15.6% of 5971 doses, mostly mild. Skin prick test wheal diameters decreased significantly (*P* < .001); s-IgE levels declined whereas s-IgG4 levels increased (*P* < .001), and sIgG4/sIgE ratios improved. No significant clinical or immunologic changes occurred during the observation period.

**Conclusions:**

Targeting a low maintenance dose of 300 mg E-OIT produces significant clinical and immunologic changes in individuals with egg allergy while maintaining low risk of adverse reactions.

Food allergy affects 6% to 10% of children worldwide, with prevalence varying by region, age, and specific allergens.^1^ Egg allergy impacts up to 6% of children in Canada^2^ and is most common among infants, with a prevalence of 9.5%, declining to 1.2% by age 6 years.^3^ Although 80% of children outgrow the allergy,^4^ accidental exposures remain common in those with persistent allergy,^5^ significantly impacting quality of life. Historically, management relied on strict avoidance and carrying epinephrine, creating a substantial burden for patients and their families.^6^^,^^7^

Egg plays a key role in childhood nutrition, is a staple in many diets, and is ubiquitously present in cooked foods, making avoidance difficult. Oral immunotherapy (OIT) offers a promising approach, introducing small, controlled doses to achieve desensitization.^8^ However, OIT is associated with frequent dose-related allergic reactions,^9^ including anaphylaxis, particularly during dose escalation.^10^ This highlights the need for optimizing OIT protocols to balance efficacy with safety.

Early egg OIT (E-OIT) studies demonstrated both potential and limitations. A 2012 study using a 2-g maintenance dose reported high dropout due to allergic reactions,^11^ and another study using 4 g reported similar adverse outcomes.^12^ In contrast, a smaller study, involving only 7 participants with a low maintenance dose of 300 mg, found fewer adverse events, with most participants tolerating 2 g of egg protein after 24 months.^13^ Similarly, a study using 194-mg maintenance doses over 12 months reported that 71.4% of OIT participants tolerated 1/32 of a whole egg after stopping treatment, compared with none in the avoidance group.^14^ Although previous studies have noted decreases in IgE and increases in IgG4 with E-OIT,^12^^,^^14^, ^15^, ^16^ these immunologic changes have not been well characterized in controlled, longitudinal low-dose protocols.

We aimed to assess the clinical and immunologic effect of a 300-mg maintenance dose of raw egg white protein in children with egg allergy.

## Methods

### Study design and participants

This randomized, open-label clinical trial was conducted at the Montreal Children’s Hospital (2018-2023) using a self-designed E-OIT protocol. Children aged 5 to 18 years with a confirmed egg allergy were eligible. To confirm persistent clinical reactivity and exclude natural tolerance, all participants were required to have a positive double-blind, placebo-controlled food challenge (DBPCFC) at screening, performed within 1 to 2 months before randomization. Diagnosis also required a positive egg skin prick test (SPT) result (wheal diameter ≥ 3 mm) and/or elevated egg-specific IgE level (sIgE ≥ 0.35kU/L).

Only those children who failed baked egg introduction were eligible; those tolerating baked egg were excluded. All enrolled participants were instructed to strictly avoid egg throughout the study. Exclusion criteria included unstable respiratory status, intercurrent active disease (eg, infectious, inflammatory conditions) at the time of starting desensitization, non–IgE-mediated reactions to egg, immunopathological conditions, current use of immunosuppressants or β-blockers, eosinophilic gastrointestinal disorders, or epinephrine contraindication. Concurrent immunotherapy to other allergens was not permitted; however, some participants had previously completed OIT for other allergens, such as milk. The study was approved by the Ethics Committee of the Montreal Children’s Hospital (Research Ethics Board number approval: 2017-3182), and written informed consent was obtained from parents or legal guardians.

### E-OIT protocol

**Fig 1:**
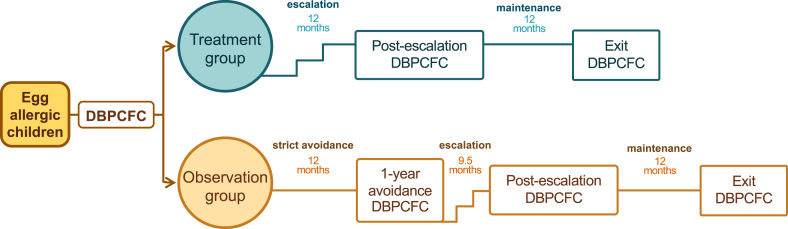
Study design. Participants with egg allergy were randomized into 2 groups: the treatment group, which began E-OIT immediately, and the observation group, which underwent 12 months of strict egg avoidance before crossing to the treatment arm. A DBPCFC was conducted to assess the CTD at 3 key time points: before start OIT (pre-OIT), at postescalation, and at exit DBPCFC. Time intervals between phases are indicated for each group to reflect differences in progression.

The trial evaluated the efficacy and immunologic effects of a low-dose E-OIT in children, targeting 300-mg daily maintenance dose of egg white protein powder (Canadian Protein, Canada). All procedures, including updosing, maintenance, and DBPCFCs, were conducted at the Center for Innovative Medicine, Montreal Children’s Hospital. Participants were randomized to either a treatment group receiving active desensitization or an observation group, following strict egg avoidance for 12 months. Afterward, the observation group underwent a DBPCFC to confirm persistent egg allergy. Only those participants who remained reactive proceeded to the treatment arm, ensuring all participants ultimately underwent desensitization therapy. The study design is illustrated in Fig 1.

Egg white protein powder was encapsulated by a licensed pharmacy (Uniprix Pharmacy, Montreal, Canada), stored at 4°C, and dispensed in preweighed capsules. Initial dosing was based on the highest tolerated amount from the screening DBPCFC. Updosing occurred every 2 weeks under medical supervision, beginning with doses as low as 0.2 mg and progressing to 300 mg. Participants were instructed to avoid all other forms of egg (cooked or processed) during treatment. Detailed stepwise dosing protocols and escalation criteria are given in Tables E1 to E3 (in the Online Repository available at www.jaci-global.org).

Dose escalation was individualized on the basis of participant tolerance. Increases were permitted only if home doses were well tolerated (as confirmed via symptom diaries and parental report), and the participant was clinically stable with normal physical examination and vital signs (eg, heart rate, blood pressure, and oxygen saturation). Participants received the new dose onsite and were monitored for at least 2 hours. Postescalation DBPCFCs were performed after 9.5 to 12 months, depending on escalation pace and scheduling. For home dosing, capsule contents were mixed with vehicle food (eg, applesauce and chocolate pudding), and taken daily at the same time. Participants were advised to avoid exercise for 2 hours before and after dosing, and to refrain from using nonsteroidal anti-inflammatory drugs within 3 hours of ingestion.

Symptom diaries were maintained throughout updosing and maintenance, reviewed at each visit to monitor for adverse events. Epinephrine was administered conservatively to ensure safety. Home-based reactions were assessed via diary entries, direct communication with the study nurse, or medical records (eg, emergency visits).

After reaching the 300-mg dose, participants continued daily treatment for 12 months while maintaining an egg-free diet. Upon completion, an exit DBPCFC was performed, and participants were then advised to reintroduce natural forms of egg into their diet at least 3 times per week.^17^

All dose escalations and food challenges were conducted under controlled conditions at the Center for Innovative Medicine to ensure safety and consistency.

### Study outcomes

The primary outcome was the proportion of participants in each group (treatment vs observation) who tolerated a cumulative dose (CTD) greater than or equal to 1000 mg of egg protein at the 12-month evaluation point. This corresponded to the postescalation DBPCFC for the treatment group and the post-avoidance DBPCFC for the observation group. Secondary outcomes included the proportion tolerating 2000 mg or higher at the exit DBPCFC (after 12 months at 300 mg), the frequency and severity of adverse events (AEs), and changes in Gal d 1 and Gal d 2 sIgE and sIgG4 levels before, during, and after the E-OIT protocol.

### SPT, blood samples, and immunologic parameters

SPTs were performed using commercially available extracts. Blood samples were collected before treatment or observation, after 12 months of avoidance (observation group), and postescalation and exit visits. Serum samples were stored at −80°C until analysis. Gal d 1 (ovomucoid) and Gal d 2 (ovalbumin) sIgE and sIgG4 levels were quantified via ELISA, as described in this article’s Methods section in the Online Repository available at www.jaci-global.org.

### Adverse events

AEs were classified using the system described by De Schryver et al,^6^ distinguishing between local reactions, nonanaphylactic allergic reactions, involving a single organ system, and anaphylactic allergic reactions, which involve multiorgan systems, each graded by severity. Anaphylaxis was defined as the involvement of 2 or more organ systems and/or the presence of hypotension.^18^ Detailed criteria and definitions are provided in this article’s Methods section in the Online Repository.

### Statistical considerations and analyses

The study was powered to detect a 50% improvement in egg tolerance (α = 0.05, power = 80%), requiring 17 participants per group (34 total). Because of recruitment constraints, 22 participants were enrolled and 20 randomized. Although this reduced power to detect small effects, the sample size was adequate to assess feasibility, safety, and preliminary efficacy.

Statistical analyses were performed using R (version 4.2.2, Vienna, Austria). Unpaired tests compared groups, whereas paired tests assessed pretreatment and posttreatment changes (*t* tests for normal data; Wilcoxon rank-sum for nonparametric data). Proportions were compared using chi-square test or Fisher exact test. Descriptive statistics summarized distributions. Multiple regression models examined associations between desensitization outcomes and baseline variables (eg, sex, age, SPT, sIgE, and sIgG4). A *P* value less than or equal to .05 was considered statistically significant in all cases.

## Results

### Participant characteristics

**Fig 2:**
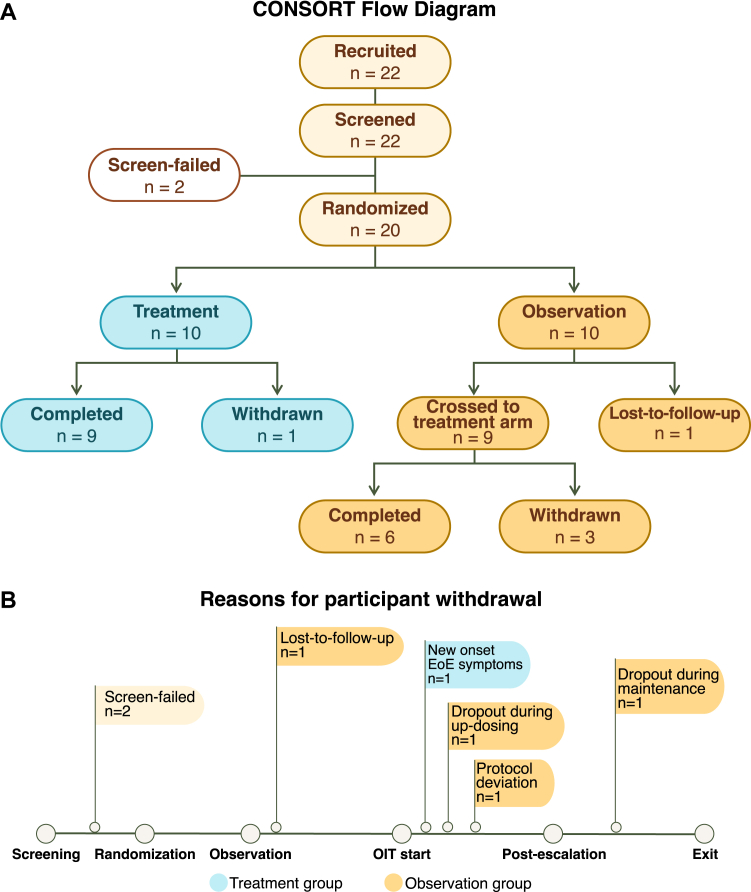
**A,** CONSORT flow diagram of the E-OIT study. A total of 22 participants with egg allergy were recruited and screened. Two were excluded because of screening failure, and 20 participants were randomized to either the treatment (n = 10) group or the observation (n = 10) group. **B,** Reasons for withdrawal are indicated along a timeline. These included allergic reactions during updosing (n = 1), allergic reactions during maintenance (n = 1), protocol deviation (n = 1), loss to follow-up (n = 1), and new-onset eosinophilic esophagitis (n = 1). In total, 15 participants completed the postescalation and exit DBPCFCs and were included for analyses. *EoE*, Eosinophilic esophagitis.

Of 22 enrolled participants, 2 failed the screening DBPCFC and were excluded. The remaining 20 were subsequently randomized: 10 to the E-OIT group, and 10 to observation (Fig 2, *A*). Five participants withdrew: 1 during updosing, 1 during maintenance, 1 because of protocol deviation, 1 lost to follow-up, and 1 because of new-onset eosinophilic esophagitis. Preexisting eosinophilic esophagitis was an exclusion criterion (Fig 2, *B*). Sixteen patients completed the escalation phase, and 15 completed the full protocol. No participants received concurrent immunotherapy during the study, though some had previously completed OIT for milk. Baseline demographics and comorbidities, such as asthma (n = 7), eczema (n = 9), and allergic rhinitis (n = 11), are detailed in Table I and were included in the regression analyses.

**Table I:** Study subject demographics and clinical characteristics of patients with egg allergy participating in E-OIT

Characteristics	Treatment (n = 10)	Observation (n = 10)
Age at baseline (y)	11 (6-13.75)	12 (9-17)
Female sex	6 (60)	6 (60)
CTD at baseline (mg)	14.1 (8.1-82.60)	20.1 (20.1-95.10)
SPT, egg extract (mm)	8.5 (5.25-12.75)	6 (5-9)
Atopic history, n (%)		
Known asthma	2 (20)	5 (50)
Known eczema	4 (40)	5 (50)
Known allergic rhinitis	5 (50)	6 (60)
	n = 9	n = 9
Gal d 2 (ovalbumin) sIgE (kU/L)	388.23 (46.62-2129.83)	949.55 (163.93-2161.49)
Gal d 2 (ovalbumin) sIgG4 (μg/mL)	507.39 (171.14-629.25)	331.88 (174.72-890.94)
Gal d 1 (ovomucoid) sIgE (kU/L)	292.74 (19.79-635.83)	171.80 (59.66-714.05)
Gal d 1 (ovomucoid) sIgG4 (μg/mL)	456.96 (206.14-614.27)	387.25 (339.89-800.68)

Values are median (IQR) or n (%). Ovalbumin and ovomucoid sIgE and sIgG4 levels were determined via ELISA.

### Desensitization outcomes

#### Low-dose E-OIT increases ability to ingest higher doses of egg protein

1. Initial randomization

At baseline, the median CTD was 14.1 mg (interquartile range [IQR], 8.1-82.60) in the treatment group (n = 10) and 20.1 mg (IQR, 20.1-95.10) in the observation group (n = 9), with no significant difference between them (*P* = 1).

**Fig 3:**
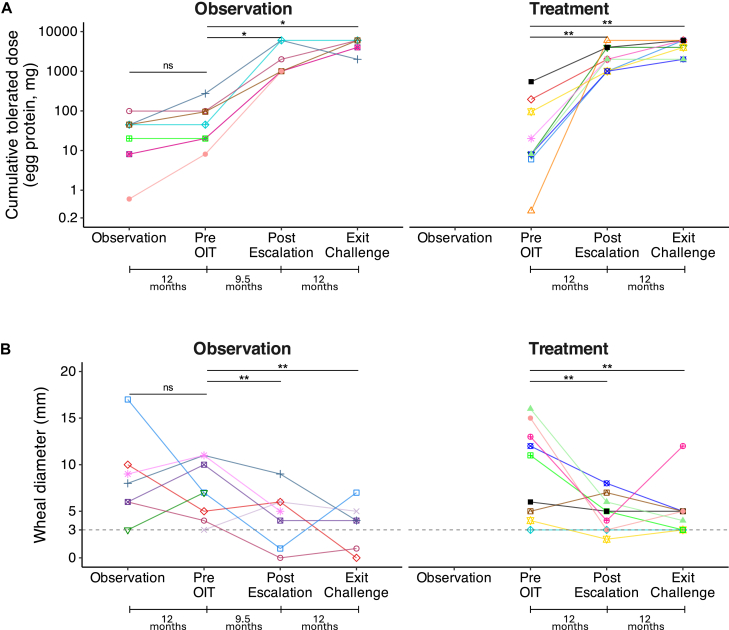
Clinical outcomes across time points in the observation and treatment groups. (**A**) CTD of egg protein (mg), as assessed by DBPCFC, and (**B**) SPT wheal diameters (mm), measured at key time points: 12 months before OIT for the observation group, and from the start of OIT to postescalation and exit DBPCFCs for all participants. Each marker shape represents a unique participant and is used for visual distinction only. The horizontal dashed line in Fig 3, *B*, indicates the SPT positivity threshold (≥3 mm). Time intervals between measurements are indicated below each facet to reflect differences in study design across groups. Statistically significant differences were assessed using the Wilcoxon signed-rank test (∗*P* < .05, ∗∗*P* < .01, ∗∗∗*P* < .001, NS: *P* > .05). *NS*, Not significant.

After 1 year of egg avoidance, the observation group underwent a DBPCFC. Their median CTD increased slightly to 32.3 mg (IQR, 8.1-44.95), which was not statistically significant (*P* = .06), and remained comparable to the baseline CTD of the treatment group (*P* = .6) (Fig 3, *A*).

In the treatment group (n = 9), the postescalation DBPCFC was performed at a median of 12 months after OIT initiation. The median CTD increased significantly from 14.1 mg to 2000 mg (IQR, 1000-4000, *P* = .004), with 100% (9 of 9) tolerating 1000 mg or more and reaching the primary end point. In contrast, at the 12-month DBPCFC following avoidance, 0% (0 of 10) of participants in the observation group tolerated 1000 mg or more (*P* < .001). After crossing over into treatment, the observation group underwent a postescalation DBPCFC at a median of 9.5 months, with their CTD increasing significantly from 20.1 mg to 1000 mg (IQR, 1000-4000; *P* = .02). This timing variation between groups was due to the individualized nature of the protocol, allowing for differences in escalation pace or participant scheduling.

Exit DBPCFCs were conducted after a median of 12 months on a stable 300-mg dose. The treatment group (n = 9) reached a median CTD of 6000 mg (IQR, 4000-6000; *P* = .009), whereas the observation group (n = 6) who had crossed over to treatment achieved a median CTD of 5000 mg (IQR, 4000-6000; *P* = .04).

2. Per-protocol analysis: Combined results from all participants

We performed a per-protocol analysis on the entire group who completed the low-dose E-OIT. At baseline (n = 19), the median CTD increased from 20.1 mg (IQR, 8.1-95.10) to 2000 mg (IQR, 1000-4000) postescalation (n = 16, *P* = .0005), and to 6000 mg (IQR, 4000-6000) at the exit DBPCFC (n = 15, *P* = .0007) (Fig 3, *A*).

At the end of the randomized phase, 100% (9 of 9) of participants in the treatment group achieved the primary end point of tolerating a cumulative dose of 1000 mg or higher at the postescalation DBPCFC. In contrast, 0% (0 of 9) of participants in the observation group reached this threshold after 1 year of egg avoidance (*P* < .001). Following the 12-month maintenance phase, 100% (15 of 15) of participants who completed the protocol tolerated 2000 mg or higher at the exit DBPCFC; among them, 80% tolerated 4000 mg or higher and 53% tolerated the full 6000-mg dose, equivalent to 1 whole egg. One participant discontinued during the maintenance phase because of persistent allergic reactions, whereas all others achieved desensitization (Fig 3, *A*).

#### Low-dose E-OIT improves skin testing results in participants with egg allergy

In the observation group, SPT wheal diameters remained unchanged during the 1-year avoidance period (7 mm; IQR, 5.75-9.25) at baseline to 7 mm (IQR, 5-10) 1 year after (*P* = 1). In the treatment group, the median baseline was 8.5 mm (IQR, 5.25-12.75). Despite the year of avoidance, there remained no significant difference in the wheal diameters between the treatment and observation groups (*P* = .4) (Fig 3, *B*).

Among participants completing E-OIT, wheal size declined significantly from a median of 7 mm (IQR, 5-11) to 5 mm (IQR, 3-6) after the escalation phase (*P* = .006), and to 4 mm (IQR, 3-5) at the exit challenge (*P* = .007) (Fig 3, *B*).

#### Low-dose E-OIT reduces egg-sIgE and elevates egg-sIgG4 concentrations in serum

1. Initial randomization

**Fig 4:**
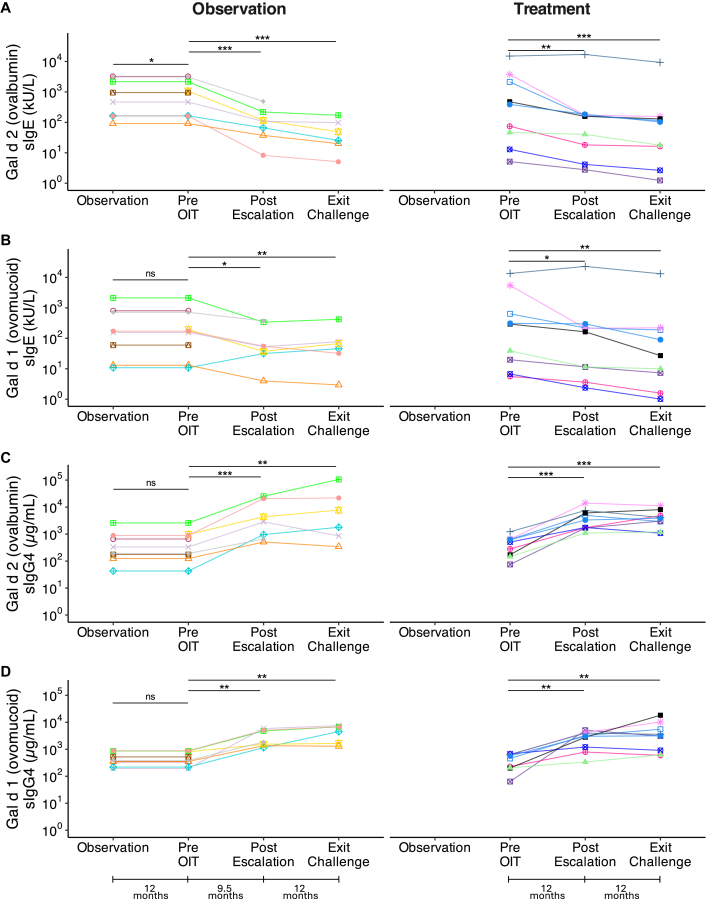
Immunologic outcomes in the treatment and observation groups. **A,** Gal d 2 (ovalbumin) sIgE, **B,** Gal d 1 (ovomucoid) sIgE, **C,** Gal d 2 (ovalbumin) sIgG4, **D,** Gal d 1 (ovomucoid) sIgG4 levels quantified by ELISA. Levels of allergen-specific antibodies were measured at multiple time points: before OIT initiation, postescalation, and at the exit DBPCFC for all participants. The observation group also had an additional measurement taken 12 months before egg avoidance. Each marker shape represents a unique participant and is used for visual distinction only. Statistically significant differences were determined by Welch *t* test (∗*P* < .05, ∗∗*P* < .01, ∗∗∗*P* < .001, NS: *P* > .05). *NS*, Not significant.

At baseline, there were no significant differences in Gal d 1 or Gal d 2 sIgE and sIgG4 levels between the treatment (n = 9) and observation groups (n = 9) (*P* > .05). After 12 months of avoidance, the observation group showed no significant changes in Gal d 1 or Gal d 2 sIgG4, and no change in Gal d 1 sIgE. However, Gal d 2 sIgE slightly increased (*P* = .01) (Fig 4).

In the treatment group (n = 9), Gal d 1 and Gal d 2 sIgE levels decreased significantly after postescalation (*P* < .01), with similar reductions in the observation group, after crossing over to the treatment arm (*P* < .01). At the exit DBPCFC, sIgE levels continued to decline significantly in both groups (*P* < .01). Conversely, Gal d 1 and Gal d 2 sIgG4 concentrations increased significantly from baseline to postescalation (*P* < .001) and continued to rise at the exit challenge (*P* < .001).

2. Per-protocol analysis: Combined results from all participants

**Fig 5:**
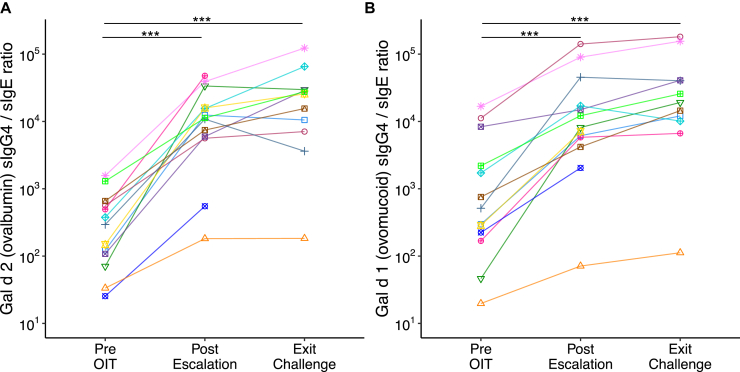
Egg component sIgG4/IgE ratios. (**A**) Gal d 2 (ovalbumin) and (**B**) Gal d 1 (ovomucoid) sIgG4/IgE ratios at the start of OIT, postescalation, and at exit DBPCFC for all participants. Each marker shape represents a unique participant and is used for visual distinction only. Statistically significant differences were assessed using the Wilcoxon signed-rank test (∗∗∗*P* < .001).

All participants completing E-OIT showed significant decreases in sIgE levels (*P* < .001) and increases in sIgG4 levels (*P* < .001) to both egg proteins (Fig 4). The sIgG4/IgE ratio demonstrated a significant improvement from baseline to OIT completion (*P* < .001) (Fig 5).

### Safety analyses

Following OIT initiation, a total of 5971 doses were administered—2935 in the treatment group and 3036 in the observation group (Table II). Only 63 doses were skipped across both groups—31 in the treatment group and 32 in the observation group—because of unrelated health issues, such as ear infections. A total of 933 food-related AEs were reported (386 in the treatment group, 547 in the observation group), resulting in an overall incidence of 15.6%. AE incidence per dose was significantly lower in the treatment group (13.2%) than in the observation group (18.0%, *P* < .0001). Local reaction rates followed a similar pattern (4.4% vs 9.1%, *P* < .0001). The most frequently reported symptoms included mild abdominal discomfort and localized oral reactions, such as facial pruritus and flushing.

**Table II:** Summary of AEs during OIT

Event type	Treatment (n = 10)	Observation (n = 10)	Total	*P* value
Total doses administered	2935	3036	5971	NA
Total AEs reported	386	547	933	NA
AE incidence (% doses)	13.2	18.0	15.6	<.0001
Local reactions	128	275	403	NA
Local reaction rate (% doses)	4.4	9.1	6.7	<.0001
In-clinic reactions				
Epinephrine use during baseline DBPCFC	9 of 10 (90%)	10 of 10 (100%)	19 (95)	1
Anaphylaxis events (updosing OIT-related)	1	2	3	NA
Epinephrine use during updosing	1	2	3	NA
Epinephrine use during postescalation DBPCFC			6 of 16 (37.5%)	NA
Epinephrine use during exit DBPCFC			3 of 15 (20%)	NA
At-home reactions				
Anaphylaxis events (home, OIT dose-related)	1	5	6	NS (*P* = .14)
Epinephrine use (home, OIT dose-related)	1	5	6	NS (*P* = .14)
Severity grading (CoFAR), median (IQR)				
Baseline DBPCFC	4 (4-4)	4 (4-4)	4 (4-4), n = 20	NS (*P* = 1)
Postescalation DBPCFC			3 (2-3), n = 16	NA
Exit DBPCFC			2 (0-3), n = 15	NA

*CoFAR*, Consortium for Food Allergy Research; *NA*, not applicable; *NS*, not significant.

Anaphylaxis, defined as involvement of 2 or more organ systems and/or hypotension, occurred during DBPCFCs and OIT escalation. At the baseline DBPCFC (n = 20), epinephrine was administered in 19 participants—9 of 10 (90%) in the treatment group and 10 of 10 (100%) in the observation group, confirming clinical reactivity in all randomized participants. Anaphylaxis during supervised updosing was rare: 1 participant in the treatment group and 1 in the observation group (with 2 separate episodes during updosing at 12 mg and 200 mg) required epinephrine. All updosing reactions occurred under observation and were managed without hospitalization (Table II).

At the postescalation DBPCFC (n = 16), epinephrine was used in 4 of 16 participants (2 of 9 [22.2%] in the treatment group and 2 of 7 [28.6%] in the former observation group). At the exit DBPCFC (n = 15), 3 of 9 (33.3%) in the treatment group required epinephrine, whereas none in the former observation group (now treated) reacted (0 of 6). The severity of DBPCFC adverse reactions declined over time, with Consortium for Food Allergy Research scores decreasing from 4 (IQR, 4-4) at baseline to 3 (IQR, 2-3) at postescalation, and 2 (IQR, 0-3) at the exit DBPCFC, indicating reduced reaction severity following low-dose E-OIT (Table II).

Outside of DBPCFCs, 6 at-home anaphylactic events occurred in response to scheduled OIT doses (5 in the observation group; 1 in the treatment group), with no significant difference between groups (*P* = .14). All events met criteria for anaphylaxis per the De Schryver classification and resolved without hospitalization (Table II).

During the 1-year egg avoidance period, 3 of 10 (30%) participants in the observation group experienced accidental allergic reactions because of unintended egg ingestion, despite strict dietary instructions. These were the only accidental exposures recorded during the study. In addition, 6 of 10 (60%) participants required epinephrine during the DBPCFC conducted after the avoidance period, with a median Consortium for Food Allergy Research grade of 4 (IQR, 3-4) (Table III).

**Table III:** AEs during the 1-y egg avoidance period in the observation group (before OIT initiation) vs the treatment group

Event type	Treatment∗ (n = 10)	Observation (n = 10)	Total	*P* value
At-home reactions during 1 y of avoidance phase (observation group only)				
Accidental allergic reactions (due to unintended egg ingestion)	NA	3 of 10 (30%)	3 (30%)	NA
In-clinic reactions at the DBPCFC 1 y after avoidance (observation group only)				
Epinephrine use during DBPCFC after 1-y avoidance	NA	6 of 10 (60%)	6 (60%)	NA
Severity grading (CoFAR), median (IQR)				
DBPCFC after 1-y avoidance	NA	4 (3-4)	4 (3-4), n = 10	NA

*CoFAR*, Consortium for Food Allergy Research; *NA*, not applicable.

∗ The treatment group did not undergo a corresponding avoidance period. Values are shown as NA where applicable.

AEs occurring during the OIT phase are summarized in Table II. Events occurring during the 1-year avoidance phase before treatment are summarized in Table III.

Linear regression models were used to assess the relationship between CTDs and levels of sIgE and sIgG4. The analysis revealed a positive and highly significant association between cumulative dose and sIgG4 levels (*P* < .001), indicating that higher IgG4 levels are linked to greater tolerance of egg protein. In contrast, sIgE levels showed a negative and significant association (*P* < .01), suggesting that elevated IgE levels are associated with reduced tolerance. In addition, Poisson regression was used to evaluate the relationship between treatment and AEs. After adjusting for atopic conditions (eg, known pollen allergy, eczema, and asthma), age, and sex, we observed that younger age, male sex, and the presence of asthma increase the risk of presenting allergic reactions during treatment (*P* < .001). Moreover, the presence of eczema was significantly associated with a higher frequency of local reactions (*P* < .05).

## Discussion

E-OIT remains relatively unexplored, particularly with low-dose protocols. Although most existing studies have used maintenance doses ranging from 1 to 6 g^19^ of egg protein, our findings suggest that desensitization can be achieved with a much lower dose of 300 mg. After approximately 10 months of escalation, all participants tolerated 1000 mg or higher, and by the end of the study, 53% (8 of 15) tolerated 6000 mg, the approximate protein content of a whole egg.^20^ This represents a substantial improvement from a median baseline CTD of 20.1 mg and may allow meaningful dietary liberalization, likely improving quality of life for participants and their families.

Desensitization was progressive and accompanied by immunologic changes consistent with a shift toward tolerance. Notably, sIgE levels declined, sIgG4 levels increased, and the sIgG4/sIgE ratio improved significantly in treated participants. These findings align with previous successful OIT studies.^21^^,^^22^ In contrast, the observation group, which avoided egg for 1 year, showed no significant immunologic changes, supporting that the observed effects were due to OIT rather than natural resolution.^23^

The safety profile and feasibility of this protocol were also notable. Of 5971 doses administered, only 1.06% were skipped, primarily because of unrelated health issues, such as mild upper respiratory tract infections. AEs occurred in 15.6% of doses, predominantly mild, including localized oral symptoms and abdominal discomfort. Anaphylaxis during updosing was rare (3 episodes), all managed on site without hospitalization. Six home-based anaphylactic reactions were reported, 5 in the observation group and 1 in the treatment group; all were dose-related and occurred after OIT initiation. Moreover, 3 participants in the observation group experienced accidental allergic reactions because of unintended egg ingestion during the 1-year avoidance period, highlighting that strict dietary avoidance does not eliminate real-world risk (Table III). These findings suggest that low-dose OIT is generally well tolerated and reduces the risk of severe allergic reactions compared with complete avoidance.

SPT results also supported desensitization, showing significant reductions in wheal diameter from baseline through postescalation and exit DBPCFCs. These objective clinical changes reinforced the immunologic findings.

Research on baked E-OIT over the past 2 decades has shown that many individuals with raw egg allergies can tolerate baked goods with egg and may benefit from incorporating it into their diets.^24^, ^25^, ^26^ However, these studies did not include control groups with strict egg avoidance periods, and so they could not conclusively demonstrate that consuming baked egg was therapeutically beneficial. In addition, unlike baked E-OIT, which relies on processed egg proteins with reduced allergenicity, our study used raw egg protein powder, containing native proteins that trigger stronger allergic responses. Despite this, all participants showed increased tolerance, and adverse reactions remained mild, suggesting that our protocol is both effective and well tolerated, even with using unmodified egg proteins. Furthermore, the use of standardized dosing with egg protein powder ensures precise administration, whereas the level of baked egg exposure can vary depending on food preparation methods.^27^ Notably, a randomized controlled trial comparing baked egg ingestion to avoidance found no significant improvement: after 6 months of intervention, 23.5% (4 of 17) of the treatment group and 33.3% (6 of 18) of the control group were able to tolerate raw egg.^28^

Our open-label, delayed-treatment design allowed all participants to undergo active treatment, while enabling a controlled comparison between OIT and avoidance, which strengthened internal validity. Although the sample size (22 participants recruited, with 15 completing the full protocol) is a limitation, it is worth noting that our sample size is larger than that in previous studies.^13^ Our findings add to the growing body of evidence supporting the efficacy of OIT, using relatively low maintenance doses.^29^ A low-dose approach may offer several advantages, including reduced costs, improved tolerability, and easier implementation in clinical practice.

Comparisons with existing higher-dose OIT trials further highlight this benefit. Palosuo et al^23^ used a 1-g dose of raw egg white powder in 50 children, achieving 44% full desensitization at 8 months, increasing to 72% at 18 months. Pérez-Rangel et al^30^ used a 2808-mg dose, achieving 94% desensitization but with AEs in 69% of patients and 31% of doses. In contrast, our 300-mg protocol achieved 100% success at 2000 mg or higher and 53% tolerance to a full egg, with a far lower AE rate (15.6%). These results suggest that low-dose OIT may provide a safer, more tolerable alternative while still producing meaningful clinical benefit.

This study is limited by its small sample size and open-label design. Timing of the postescalation DBPCFC differed slightly between groups because of individualized updosing. In addition, long-term tolerance after treatment was not assessed.

Given that PALFORZIA^31^ remains the only Food and Drug Administration–approved OIT product (for peanut allergy), our findings highlight the potential of low-dose E-OIT as safer, scalable alternative for children with egg allergy. Larger studies, long-term follow-up, and mechanistic research are warranted to confirm durability and optimize dosing strategies.

**Key messages**

• **Participants taking low doses of egg over a median of 22 months successfully increased their tolerance to egg protein, with the median CTD significantly rising from 20.1 mg at baseline to 6000 mg (∼1 egg) by the end of the study.**
• **E-OIT led to a significant decrease in sIgE levels and increase in sIgG4 levels, as well as higher IgG4/IgE ratio, indicating successful desensitization.**
• **The observation group showed no significant changes in most immunologic markers except for a slight increase in ovalbumin-sIgE during the 1-year avoidance period, whereas OIT resulted in clear and significant immunologic changes.**
• **AEs were mild and infrequent, with symptoms limited to minor gastrointestinal discomfort and localized reactions, confirming the enhanced safety of the low-dose E-OIT protocol.**

## Disclosure statement

This work was supported by the Montreal Children’s 10.13039/501100022917Hospital Foundation and the 10.13039/100014131McGill University Health Centre Foundation.

Disclosure of potential conflict of interest: The authors declare that they have no relevant conflicts of interest.
