# Supplementary material for: Egg desensitization is achieved effectively and safely through a low maintenance dose protocol
Source: J Allergy Clin Immunol Glob. 2025 Jul 31;4(4):100547. doi: 10.1016/j.jacig.2025.100547 (PMC12446774; doi:10.1016/j.jacig.2025.100547)
Supplement: Supplementary Tables [file mmc1.docx]

**Supplemental material**

**Methods**

***Adverse Events***

Adverse events (AEs) were classified using the system described by De Schryver S, et al.^6^, which distinguishes between non-anaphylactic allergic reactions (AR), involving a single organ system, and anaphylactic allergic reactions (AAR), which involve multi organ systems. Local reactions, such as oral pruritus or tingling, were documented separately, while systemic reactions were classified as either AR or AAR. ARs were graded into mild, moderate and severe reactions. Mild ARs included symptoms like pruritus, urticaria, flushing or rhinoconjunctivitis. Moderate ARs involved angioedema, throat tightness, gastrointestinal complaints, or breathing difficulties (e.g. cough or rhinorrhea) other than wheezing. Severe ARs were characterized by wheezing or cyanosis.

AARs were similarly divided into mild, moderate, and severe categories. Mild AARs presented with symptoms involving skin and subcutaneous reactions (e.g., urticaria, erythema, or angioedema), gastrointestinal symptoms (e.g., oral pruritus, nausea, or mild abdominal pain), respiratory symptoms (e.g., nasal congestion, sneezing, rhinorrhea, or throat tightness), cardiovascular signs such as tachycardia, or neurological symptoms such as anxiety or changes in activity level. Moderate AARs included the presence of any of these symptoms along with crampy abdominal pain, diarrhea, recurrent vomiting, dyspnea, stridor, cough, wheezing, or light-headedness. Severe AARs were defined by more critical symptoms, including loss of bowel control, cyanosis, hypoxia (oxygen saturation <92%), respiratory arrest, hypotension, dysrhythmia, severe bradycardia, cardiac arrest, confusion, or loss of consciousness. Anaphylaxis was defined as the involvement of two or more organ systems and/or the presence of hypotension^17^.

***Determination of specific IgE and IgG4 responses***

Immunologic responses, including Gal d 1 (ovomucoid) and Gal d 2 (ovalbumin) specific IgE (sIgE) and IgG4 (sIgG4) levels, were quantified by using egg-specific enzyme-linked immunosorbent assay (ELISA). Polystyrene 96-well half-area microplates (Corning, 3690) were coated overnight at 4°C with either Gal d 1 (ovomucoid) (Innovative Research, IEGGTI500MG) or Gal d 2 (ovalbumin) proteins (Sigma, A5503-5G) (2 µg/ml, 50 µL/well), along with capture antibodies: goat anti-human IgE (BETHYL, A80-108A) (1:1000, 50µL/well) or anti-human IgG4 (Invitrogen, BMS2095) (1:250, 50µL/well), respectively. Participant serum samples were diluted at various dilution factors (1:10 – 1:10,000) and human IgE or IgG4 standards were serially diluted to create eight consecutive doubling dilutions. Plates were then incubated with the standards and participant serum samples for two hours (50 µL/well, RT). Detection used biotinylated goat anti-human IgE detection antibody (BETHYL, A80-108B) (1:20,000, 50 µL/well) followed by HRP-streptavidin (Bio Legend, 405210) (1:3,000, 50 µL/well) or HRP-Conjugate anti-human IgG4 monoclonal antibody (Invitrogen, BMS2095) (1:200, 50 µL/well) and TMB substrate (Invitrogen, 00-42-1-56, 50µL/well). Optical density (OD) was measured at 450 nm with a 570 nm reference. A 4-parameter logistic (4PL) standard curve was generated, and all samples were analyzed in duplicate for accuracy.

**Supplemental Table 1.** DBPCFC dosing protocol at baseline. Double-blind, placebo-controlled, food challenge dosing protocol up to a cumulative dose of 845.1 mg of raw egg protein or placebo.

| **Step** | **Dose (mg)** | **Time before next dose** | **Cumulative tolerated dose (mg)** | **Notes** |
| --- | --- | --- | --- | --- |
| 1 | 0.2 | 30 min | 0.2 | Start dose; observe for symptoms |
| 2 | 0.4 | 30 min | 0.6 | Proceed if no reaction |
| 3 | 1.5 | 30 min | 2.1 |  |
| 4 | 6 | 30 min | 8.1 |  |
| 5 | 12 | 30 min | 20.1 |  |
| 6 | 25 | 30 min | 45.1 |  |
| 7 | 50 | 30 min | 95.1 |  |
| 8 | 100 | 30 min | 195.1 |  |
| 9 | 150 | 30 min | 345.1 |  |
| 10 | 200 | 30 min | 545.1 |  |
| 11 | 300 | Final dose | 845.1 | Observe ≥ 2 hours after last dose |

**Supplemental Table 1.** DBPCFC dosing protocol used at baseline. Participants received incremental doses of raw egg protein or placebo every 30 minutes, up to a maximum cumulative dose of 854.1 mg. Progression to the next dose occurred only if no objective allergic symptoms were observed. Following the final dose, participants were monitored for a minimum of 2 hours. This cumulative dose represents the total egg protein administered within a single day.

**Supplemental Table 2.** Escalation phase STEP protocol.

| Step | Dose (mg) | Duration | Conditions for escalation | Observation protocol | Adverse reaction management |
| --- | --- | --- | --- | --- | --- |
| 1 | 0.2 | 14 days | - Dose increased only if previous dose was well tolerated, participant is clinically stable, and vitals are normal. - Assessment includes symptom diary review, physical exam, and pre-/post-dose vitals. | - All dose escalations are at the research unit with ≥ 2 hours of observation.      - 2 hours under medical supervision at the clinic - Vitals pre and post dose | - See Supplemental Table 2A for grading and management of mild, moderate and severe reactions. - Mild reactions: Treated symptomatically (antihistamine), dose continued if asymptomatic. - Moderate reactions: Dose paused, restarted at previously tolerated level next day. - Severe reactions: Treated immediately, dose reduced to 1/10, or protocol reassessed. |
| 2 | 0.4 | 14 days |  |  |  |
| 3 | 0.8 | 14 days |  |  |  |
| 4 | 1.5 | 14 days |  |  |  |
| 5 | 3 | 14 days |  |  |  |
| 6 | 6 | 14 days |  |  |  |
| 7 | 12 | 14 days |  |  |  |
| 8 | 25 | 14 days |  |  |  |
| 9 | 50 | 14 days |  |  |  |
| 10 | 75 | 14 days |  |  |  |
| 11 | 100 | 14 days |  |  |  |
| 12 | 125 | 14 days |  |  |  |
| 13 | 150 | 14 days |  |  |  |
| 14 | 200 | 14 days |  |  |  |
| 15 | 250 | 14 days |  |  |  |
| 16 | 300 | Maintenance (~12 months) | Final dose tolerated |  | Continued unless major adverse events |

**Supplemental Table 2.** Low-dose egg OIT escalation protocol. Each dose was administered daily for 14 days before escalation, with progression permitted only if the previous dose was well tolerated and safety criteria were met. Dose increases were conducted at the clinical research unit under direct medical supervision, with a minimum 2-hours observation period. Adverse reactions were graded and managed according to predefined criteria (see Supplemental Table 2A). The final tolerated dose of 300 mg was maintained daily for approximately 12 months unless significant adverse events occurred.

**Supplemental Table 2A.** Grading and management of adverse reactions during OIT dose escalation.

| **Severity** | **Symptoms** | **Action** |
| --- | --- | --- |
| Mild | Pruritus,  Urticaria,  Flushing,  Rhino conjunctivitis | Observe and monitor the participant;, administer an oral antihistamine (e.g., rupatadine or cetirizine) if needed. Notify the research team, who will assess whether dose escalation should proceed or be suspended. |
| Moderate | Angioedema,  Throat tightness,  Gastrointestinal complaints  (cramping, pain, vomiting, diarrhea)  Respiratory symptoms (Cough,  Mucous production) | Administer intramuscular epinephrine as needed; along with antihistamines as prescribed. Seek urgent medical care and notify the research team. Next dose to be administered under supervision at the CIM. |
| Severe | Wheeze, Respiratory Distress  Hypoxia,  Cyanosis,  Hypotension  Circulatory collapse (Shock) | Administer intramuscular epinephrine immediately; call emergency services (911) if needed. Seek emergency care without delay and contact research team. If symptoms persist beyond 10 minutes, a second epinephrine dose may be advised. |

**Supplemental Table 2A.** Grading and management of adverse reactions during OIT dose escalation. Reactions were categorized as mild, moderate, or severe based on symptom type and clinical severity. Corresponding interventions ranged from observation and antihistamine administration to intramuscular epinephrine and emergency care. Decisions regarding continuation, dose adjustment, or suspension were made in consultation with the research team, following in-clinic assessment protocols.

**Supplemental Table 3.** Dosing protocol at **post-escalation and exit DPBCFC**. Double-blind, placebo-controlled, food challenge dosing protocol up to a cumulative dose of 6000 mg of raw egg protein or placebo.

| **Step** | **Dose (mg)** | **Time before next dose** | **Cumulative tolerated dose (mg)** | **Notes** |
| --- | --- | --- | --- | --- |
| 1 | 100 | 30 min | 100 | Start dose; observe for symptoms |
| 2 | 300 | 30 min | 400 | Proceed if no reaction |
| 3 | 600 | 30 min | 1000 |  |
| 4 | 1000 | 30 min | 2000 |  |
| 5 | 2000 | 30 min | 4000 |  |
| 6 | 2000 | Final dose | 6000 | Observe ≥ 2 hours after last dose |

**Supplemental Table 3:** Dosing protocol at post-escalation and exit DBPCFC. Participants received incremental doses of raw egg protein or placebo every 30 minutes, up to a cumulative dose of 6000 mg. Each step proceeded only if no objective symptoms occurred, and participants were observed for a minimum of 2 hours following the final dose. The cumulative dose represents the total egg protein administered within a single day. This protocol was used to assess desensitization and clinical response after low-dose egg OIT.
